# circPTCH1 promotes invasion and metastasis in renal cell carcinoma via regulating miR-485-5p/MMP14 axis

**DOI:** 10.7150/thno.47239

**Published:** 2020-08-29

**Authors:** Huan Liu, Guanghui Hu, Zaoyu Wang, Qunlong Liu, Jin Zhang, Yonghui Chen, Yiran Huang, Wei Xue, Yunfei Xu, Wei Zhai

**Affiliations:** 1 Department of Urology, Shanghai Tenth People's Hospital, School of Medicine in Tongji University, Shanghai 200072, China.; 2 Department of Pathology, Renji Hospital, School of Medicine in Shanghai Jiao Tong University, Shanghai 200127, China.; 3 Department of Urology, Shanghai Tenth People's Hospital, Nanjing Medical University, Nanjing 210029, China.; 4 Department of Urology, Renji Hospital, School of Medicine in Shanghai Jiao Tong University, Shanghai 200127, China.

**Keywords:** circPTCH1, miR-485-5p, MMP14, metastasis, renal cell carcinoma

## Abstract

**Background:** Circular RNAs (circRNAs) are a new class of non-coding RNAs (ncRNAs) that are derived from exons or introns by special selective shearing. circRNAs have been shown to play critical roles in various human cancers. However, their roles in renal cell carcinoma (RCC) and the underlying mechanisms remain largely unknown.

**Methods:** A novel circRNA-circPTCH1, was identified from a microarray analysis of five paired RCC tissues. Then, we validated its expression and characterization through qRT-PCR, gel electrophoresis, RNase R digestion assays and Sanger sequencing. Functional experiments were performed to determine the effect of circPTCH1 on RCC progression both *in vitro* and *in vivo*. The interactions between circPTCH1 and miR-485-5p were clarified by RNA pull-down, luciferase reporter and RNA immunoprecipitation (RIP) assays.

**Results:** We observed that circPTCH1 was up-regulated in RCC cell lines and tumor samples, and higher levels of circPTCH1 were significantly correlated with worse patient survival, advanced Fuhrman grade and greater risk of metastases. Elevated circPTCH1 expression led to increased migration and invasion of RCC cells both *in vitro* and *in vivo* whereas silencing circPTCH1 decreased migration and invasion and impeded the epithelial-mesenchymal transition (EMT) of RCC cells. Mechanistically, we elucidated that circPTCH1 could directly bind miR-485-5p and subsequently suppress expression of the target gene MMP14.

**Conclusion:** circPTCH1 promotes RCC metastasis *via* the miR-485-5p/MMP14 axis and activation of the EMT process. Targeting circPTCH1 may represent a promising therapeutic strategy for metastatic RCC.

## Introduction

Renal cell carcinoma (RCC) is one of the most lethal malignancies in the world and is estimated to account for approximately 4% (73,750 new cases) of newly diagnosed carcinomas and 2% (14,830 deaths) of cancer deaths in United States in 2020 1. Localized or early stage RCCs can be addressed with surgical treatments such as partial or radical nephrectomy, which has a 93% five-year survival rate 2. Although advances in imaging technologies have improved the early diagnosis rate of RCC 3, 20-30% RCC patients still present with evidence of distant metastases during their initial treatment 4. Further, for those patients who have surgery, there is a 50% risk of developing metastases in the future 5. Among all the renal cancer-related deaths, more than 90% are associated with RCC metastasis 6. Though the combination of tyrosine kinase inhibitors (TKIs) and immune checkpoint inhibitors (ICIs) has recently been recommended as a front-line therapy in metastatic RCC and has improved patient prognosis 7, most patients will still experience tumor progression and ultimately die due to drug resistance 8, 9. Thus, it is of great clinical significance to clarify the underlying mechanisms of RCC metastasis and to identify additional prognostic biomarkers and therapeutic targets for metastatic RCC.

Circular RNAs (circRNAs) are a new class of non-coding RNAs (ncRNAs) that generated from exons or introns through special selective shearing and have a covalently closed loop structure without terminal 5′ caps and 3′ poly A tails 10, 11. Due to this specific closed loop structure, circRNAs exhibit greater stability than linear RNAs under the degradation of exonuclease RNase R 12. circRNAs were initially regarded as by-products of splicing errors when discovered many years ago. However, the wide use of high-throughput sequencing and bioinformatics analysis has demonstrated that circRNAs are endogenous, abundant, and conserved in mammalian cells which suggest that they likely have specific biological functions 13. Recent studies have reported various physiological functions of circRNAs. They can function as protein-coding genes, sponging microRNA (miRNA/miR) to hamper mRNA translation or binding with RNA-associated proteins. For example, circFoxo3 arrests the function of CDK2 and retards cell cycle by forming a circFoxo3- p21-CDK2 triplex 14. Circ0072391 promotes hepatoblastoma cell proliferation and apoptosis by sponging miR-503-5p 15. A 174 amino acid (aa) novel protein encoded by circAKT3 competitively interacts with phosphorylated PDK1 to suppress the PI3K/AKT signal intensity in glioblastoma 16.

MicroRNAs, a class of short (19-22nt) ncRNAs, regulate target gene expression through targeting 3'untranslated (UTR) regions and preventing mRNA translation 17. In recent studies, miR-485-5p has been demonstrated to act as a tumor suppressor in various cancers. For instance, miR-485-5p inhibits the development of hepatocellular carcinoma by downregulating WBP2 and blockading the Wnt/β-catenin signaling pathway 18. In gliomas, miR-485-5p suppresses tumor cell proliferation via directly targeting paired box 3 (PAX3) 19. However, to our knowledge, the role of miR-485-5p in RCC has not been described to date.

In this research, we evaluated the expression profiles of circRNAs in metastatic RCC tissues and identified a novel circRNA generated from the patched-1 (PTCH1) gene, termed circPTCH1. PTCH1 is a key component of the hedgehog (HH) signaling pathway. Mutational inactivation of PTCH1 could lead to aberrant activation of the HH pathway, which is involved in the initiation, acceleration, metastasis, and therapeutic resistance of a large number of tumors 20, 21. Here, we found that this PTCH1-derived circRNA was significantly up-regulated in tumor tissues and cell lines using microarray analysis and qRT-PCR assays. Higher circPTCH1 expression was correlated with worse patient survival, advanced RCC Fuhrman grade and more metastases. Functional assays revealed that circPTCH1 promotes migration and invasion of RCC cells both *in vitro* and *in vivo*. Mechanistic studies implied that circPTCH1 enforces MMP14 expression by competitively binding to miR-485-5p and inducing the epithelial-mesenchymal transition (EMT) process. Therefore, our findings indicate that circPTCH1 may represent a promising therapeutic target to inhibit the invasion and metastases of RCC.

## Materials and Methods

### Human RCC tissue samples

The five snap-frozen tumor tissues samples used for circRNA microarray analysis were collected from metastatic RCC patients that underwent nephrectomy at the Department of Urology of Shanghai Tenth People's Hospital and Shanghai Renji Hospital. Furthermore, 39 RCC tissues and matched adjacent normal samples between January 2017 and June 2019 were collected from Shanghai Tenth People's Hospital and Renji Hospital for validation. All RCC cases were confirmed by a senior pathologist from each hospital. The demographic data, pathologic characterization and representative pathological figures of RCC samples are listed in Table S1. The use of all tissue specimens was evaluated and approved by the Ethical Committees of Shanghai Tenth People's Hospital affiliated Tongji University, and Renji Hospital affiliated School of Medicine in Shanghai Jiaotong University. Written informed consent was obtained from every patient.

### Microarray analysis

Total RNA from each sample was quantified using a NanoDrop ND-1000. The sample preparation and microarray hybridization were performed based on Arraystar's standard protocols (KANGCHEN Biotech, Shanghai, China). In brief, total RNA was digested with RNase R (Epicentre, Inc.) to remove linear RNAs and enrich for circular RNAs. Then, the enriched circRNAs were amplified and transcribed into fluorescent cRNA using a random priming method (Arraystar Super RNA Labeling Kit; Arraystar). The labeled cRNAs were hybridized onto the Arraystar Human circRNA Array V2 (8×15K, Arraystar). Next, the slides were washed and the arrays were scanned using an Agilent Scanner G2505C. Agilent Feature Extraction software (version 11.0.1.1) was applied to analyze the acquired array images. Quantile normalization and subsequent data processing were performed using the R software limma package. Differentially expressed circRNAs with statistical significance between two groups were identified through Volcano Plot filtering. Significantly differentially expressed circRNAs were then further filtered through analysis of Fold Change. Hierarchical Clustering was performed to highlight distinguishable circRNAs expression patterns among samples.

### Bioinformatics analyses

The sequence of circPTCH1 was acquired from circBase. To predict the potential miRNAs binding with circPTCH1, we used three online analysis tools: Circular RNA Interactome, circAtlas 2.0 and starBase v2.0. The expression of miRNAs and mRNAs candidates was evaluated using The Cancer Genome Atlas Program (TCGA) Kidney Clear Cell Carcinoma (KIRC) database, or the UALCAN database 22. The downstream target genes of miR-485-5p were predicted using TargetScan, starBase v2.0, miRDB, miWALK and miDIP. The websites linking to the above online databases are listed in Table S2.

### Cell lines and cultivate

Human RCC cell lines ACHN, OS-RC-2, A498, 786-O and normal kidney tubular epithelial cell HK-2 were purchased from the Cell Bank of Chinese Academy of Sciences (Shanghai, China). HK-2 was cultivated in Keratinocyte Medium (ScienCell, San Diego, USA) plus 1% Keratinocyte Growth Supplement (ScienCell, San Diego, USA) and the other RCC cells and HEK293T were cultured in Gibco Dulbecco's Modified Eagle's Medium with 10% Fetal Bovine Serum (FBS, Hyclone, Utah, USA). All media contained 1% Gibco Penicillin/Streptomycin (New York, USA). All cells were cultured in the same humidified atmosphere (37 °C with 5% CO_2_).

### RNA extraction, reverse transcription, and quantitative real-time PCR analysis (qRT-PCR)

Total RNA was extracted from cells or human tissues using Trizol reagent as directed by the manufacturer (Invitrogen, CA, USA). To isolate RNA from the nuclear and cytoplasmic fractions, a PARIS™ Kit was used (Invitrogen, CA, USA). A Nanodrop 2000 spectrophotometer was used to measure RNA purity and concentration (Thermo Fisher Scientific, Inc., Waltham, MA, USA). Reverse transcription was performed to synthesize cDNA using the PrimeScript RT reagent kit (TaKaRa, Japan). qRT-PCR was conducted using a SYBR Green qPCR Kit (Takara, Japan) and the ABI Prism 7500 Detection System (Applied Biosystems, USA). The expression of circRNAs, mRNAs and miRNAs were calculated normalized to internal control GAPDH or U6 using the 2^-ΔΔCt^ method. The detailed primer sequences are listed in Table S3.

### Western blot analysis

Cells were lysed on ice using RIPA buffer (Beyotime, Shanghai, China) containing protease inhibitors. Equal amounts (30 µg) of protein samples was loaded onto a 10% SDS-PAGE gel and then transferred to nitrocellulose or polyvinylidene fluoride membranes. The membranes were blocked in 5% skim milk and then incubated overnight at 4 °C with primary antibodies as follow: MMP14 (1:1000, Abclonal, China), E-cadherin (1:1000, Abclonal, China), N-cadherin (1:1000, Abclonal, China), Vimentin (1:1000, Abclonal, China), GAPDH (1:5000, Abcam, UK). Next, the membranes were incubated with secondary mouse or rabbit antibodies for 1 h at room temperature. After 3 washes with PBST, the fluorescence was measured using an Odyssey scanner (LI-COR, Biosciences, NE, USA).

### Immunofluorescence

After various treatments, cells were fixed with 4% paraformaldehyde, permeabilized in 0.3% Triton X-100, and then blocked in 5% goat serum. Later, cells were incubated overnight at 4 °C with antibody against E-cadherin (1:200, Abclonal, China) or N-cadherin (1:200, Abclonal, China). Secondary fluorescent antibody conjugated Alexa Fluor 488/594 was added and incubated at room temperature for 1 h. DAPI was used for nuclear counterstaining. Images were captured using a confocal microscope (Leica Microsystems, Buffalo Grove, USA).

### Wound healing assays

After various treatments, cells were seeded in triplicate into 6-well plates and, after reaching 80-90% confluency, a wound was made using a 200 μL pipette tip on the cell monolayer. Culture media was then replaced with 2% FBS DMEM. Next, cells were incubated to allow to heal for additional 24 h and then photographed via microscopy (Leica Microsystems, Mannheim, Germany).

### Transwell assays

Transwell chambers (Corning, MA, USA) with 8 μm pore size polycarbonate filters were used to evaluate the migration and invasion abilities of RCC cells. 5×10^4^ cells in serum-free media were seeded in the upper chamber pre-covered with or without Matrigel (Corning, BD356230, USA), and 600 μL 10% FBS culture media was added to the lower chamber. After incubation, cells in the upper surface of the filters were gently removed using a cotton swab. Then, the cells that migrated or invaded to the lower surface of the filter were fixed in 95% ethanol for 15 min and stained with 0.1% crystal violet solution for 20 min. Cell numbers were measured and averaged across five randomly selected fields under a microscope (Leica Microsystems, Mannheim, Germany).

### RNase R and Actinomycin D treatment assay

According to the protocols previously described 23, 24, RNA was incubated with RNase (3 units per μg, Geneseed Biotech, Guangzhou, China) for 30 min and RCC cells were exposed to 2 μg/mL Actinomycin D (HY-17559, Medchemexpress, Monmouth Junction, NJ, USA) for 8, 16, or 24 h. Then, the expression of circPTCH1 and PTCH1 were detected by qRT-PCR.

### Fluorescence *in situ* hybridization (FISH)

Cy3-labeled probe specific to cirPTCH1 was purchased from Ribobio (Guangzhou, China). Cell nuclei were stained with DAPI. The FISH experiment was performed using the Fluorescence *in situ* hybridization kit (C10910, Ribobio, Guangzhou, China) following the manufacturer instructions. Images were acquired using a microscope (Leica Microsystems, Mannheim, Germany).

### Cells transfection

Two small-interfering RNAs (siRNAs) specifically targeting circPTCH1 were designed and generated by IBSBIO Biotech (Shanghai, China). MiRNA negative control (mi-NC), miR-485-5p mimics and inhibitors were purchased from Ribobio (Guangzhou, China). Transient transfection of these reagents was conducted using Lipofectamine 3000 (Thermo Fisher Scientific). For circRNA overexpression, the circPTCH1 overexpressed plasmid was synthesized by BioLink (Shanghai, China). Then, we transfected the plasmids into HEK293T cells to package lentivirus using a Lentivirus-Packaging kit (BioLink, Shanghai, China). After 24h, lentivirus supernatants were collected and used to infect cells.

### RNA pull-down assay

The biotin-labeled circPTCH1 probe was synthesized by BIOFAVOR Biotech (Wuhan, China). In brief, 2×10^7^ cells were harvested and lysed in 100 μL RIP lysis buffer on ice, then incubated with a high-affinity biotin-labeled probe for 1 h at room temperature. Next, the suspension and streptavidin magnetic beads were mixed for 1 h at room temperature. The beads were washed using RIP wash buffer and the RNAs pulled down on the beads were extracted using Trizol and analyzed by qRT-PCR assay and gel electrophoresis.

### Luciferase reporter analysis

The potential binding sites of miR-485-5p and circPTCH1 or MMP14 were obtained from circAtlas and TargetScan, and the sequences were mutated and cloned into a psiCHECK-2 vector (Promega Corporation, WI, USA). RCC cells were seeded in 12-well plates and co-transfected with the luciferase reporter vector (circPTCH1-WT/Mut or MMP14-WT/Mut) and miR-485-5p mimics or NC. After 48 h of transfection, the relative luciferase activity was measured by Dual Luciferase Assay System according to the manufacturer's protocol (Promega, Massachusetts, USA).

### RNA immunoprecipitation (RIP) assay

The RIP assay was performed using an EZ-Magna RIP kit (Millipore, MA, USA) per the manufacturer's instructions. Briefly, RCC cells were harvested and lysed in RIP lysis buffer, and then incubated with magnetic beads coated with anti-Ago2 or anti-IgG antibody (Santa Cruz). Next, the immunoprecipitated RNAs were extracted as described above and detected by qRT-PCR.

### Orthotopic tumor implantation in nude mice

For *in vivo* tumor studies, 4-6 weeks old Balb/c nude mice were purchased from Shanghai Sipper-BK Laboratory Animal Company (Shanghai, China). The mice were kept in a specific pathogen-free environment and all operations on mice were conducted following protocols approved by the Animal Research Ethics Committee of the Shanghai Tenth People's Hospital, Tongji University. OS-RC-2 cell line stably expressing firefly luciferase cell line (OS-RC-2-luci) was constructed as previously described 9. Each mouse was injected with 1×10^6^ OS-RC-2-luci cells (vector or OE-circPTCH1) into the left subrenal capsule (1:2 mixed with Matrigel before injection). To detect the role of miR-485-5p *in vivo*, the miR-485-5p agomir or NC (Ribobio, Guangzhou, China) were injected continuously into the tail vein for 2 weeks according to prior publications and the manufacturer's recommendations 25. Tumor progress and metastasis were observed using the IVIS imaging system (Calipers, Hopkinton, USA). After 6 weeks, all mice were sacrificed and the tumors and metastatic tissues were harvested for analysis.

### Immunohistochemistry (IHC)

IHC was conducted on tumor samples from xenograft mice following the methods described previously 26. The tumor specimens were fixed in formalin, embedded in paraffin and then cut into 4 μm slices. After dewaxing, rehydration, and antigen retrieval, these slides were incubated with specific primary antibodies against MMP14, E-cadherin, or N-cadherin (Abclonal, Wuhan, China). Images were photographed using a microscope (Leica Microsystems, Mannheim, Germany).

### Statistical analysis

Statistical analyses were performed using the GraphPad Prism 8 (GraphPad Software, San Diego, CA, USA) and SPSS 19.0 software (SPSS, Inc., Chicago, USA). All data were presented as mean ± standard deviation (SD) from three independent experiments. Statistical significance was analyzed using Student's *t*-tests. The Kaplan-Meier method was applied to assess survival curves, and differences were measured by a log-rank test. The correlations between circPTCH1, miR-485-5p and MMP14 were evaluated using Pearson's correlation coefficient. All data were considered statistically significant at a *p*-value < 0.05.

## Results

### Identification of circPTCH1 from microarray analysis and characterization in RCC cells

A microarray-based circRNA expression profile analysis was conducted on five metastatic RCC tissues and paired adjacent normal specimens. In total, 9,591 distinct circRNAs were detected (Figure S1A). circRNAs with | fold change (FC) | ≥ 2 and *p*-values < 0.05 were considered to be significantly differentially expressed. The significantly dysregulated circRNAs were presented in a volcano and clustered heat map (Figure S1B-C).

**Figure 1:**
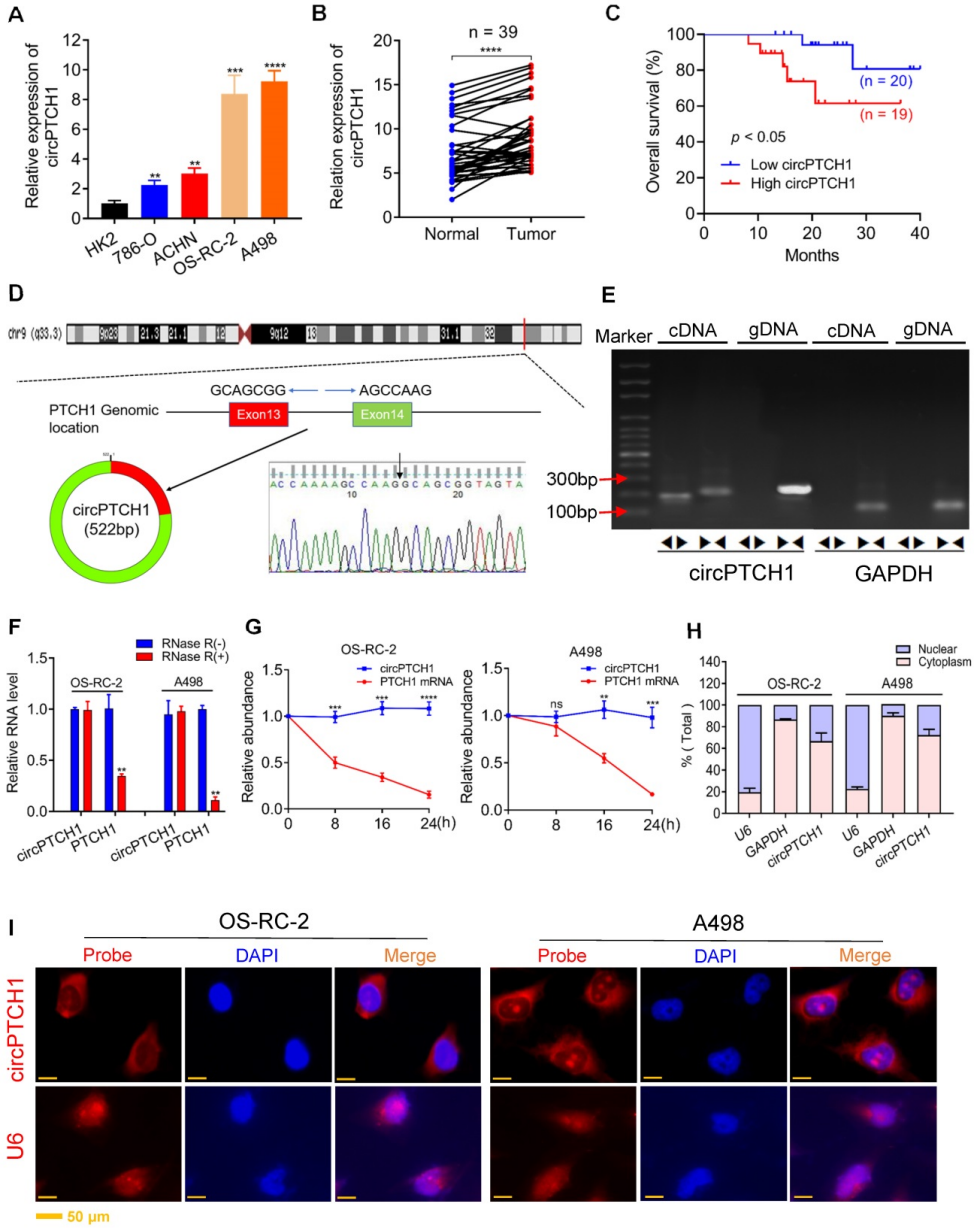
** The expression and characterization of circPTCH1 in RCC cells. A.** Relative expression of circPTCH1 in human normal kidney tubular epithelial cell HK-2 and RCC cell lines (786-O, ACHN, OS-RC-2 and A498). **B.** Relative expression of circPTCH1 detected by qRT-PCR in 39 paired ccRCC tissues compared with adjacent normal tissues. **C.** Kaplan-Meier's survival analysis indicated that circPTCH1 expression was correlated with worse patient prognosis. **D.** Schematic illustration shows that circPTCH1 formed from exon 13 and 14 of PTCH1 and the existence of circPTCH1 was validated by Sanger sequencing. Black arrow represents the back-splicing site of circPTCH1. **E.** The existence of circPTCH1 was validated by qRT-PCR and Gel electrophoresis. Divergent primers could amplify circPTCH1 in cDNA but not gDNA. GAPDH was used as negative control. **F**-**G.** The qRT-PCR results showed circPTCH1 was more stable than linear PTCH1 after treatment with RNase R or Actinomycin D. **H.** qRT-PCR analysis of circPTCH1 was conducted in nuclear and cytoplasmic fractions of OS-RC-2 and A498 cells. **I.** FISH indicated that circPTCH1 was predominantly located in cytoplasm of OS-RC-2 and A498 cells. Nuclei were stained with DAPI and circPTCH1 was labeled with Cy3. U6 probe was applied as negative control. Scale bar, 50 µm. *p < 0.05, **p < 0.01, ***p < 0.001, ****p < 0.0001. cDNA: complementary DNA; gDNA: genomic DNA; FISH: fluorescence *in situ* hybridization. RCC: renal cell carcinoma.

To identify circRNAs that promote metastasis in RCC, we focused on the top five up-regulated circRNAs according to FC (Table S4). The expression of those circRNAs was examined in both RCC cell lines and 39 paired tumor samples by qRT-PCR analysis. Among them, only hsa_circ_0139402 was found to be highly expressed in all RCC cell lines compared with HK-2 (Figure S2 and Figure 1A), which was consistent with the results of our microarray analysis. Further, hsa_circ_0139402 was also expressed at higher levels in tumor specimens compared with adjacent normal tissues (Figure 1B). Higher circPTCH1 expression was also correlated with worse RCC patient survival (Figure 1C). Because the OS-RC-2 and A498 cell lines had higher expression of circPTCH1, we selected these two cells for further studies.

Hsa_circ_0139402 consists of exon 13 and 14 of PTCH1 (522 bp) and its head-to-tail splicing structure was confirmed by Sanger sequencing (Figure 1D). Since hsa_circ_0139402 was derived from the host gene PTCH1 (Gene ID: 5727), we named it circPTCH1. Divergent and convergent primers were designed to separately detect the expression of circPTCH1 and linear PTCH1 using gel electrophoresis. We found that circPTCH1 could only be amplified in cDNA but not in genomic DNA (gDNA) using divergent primers while PTCH1 was amplified in both cDNA and gDNA by convergent primers (Figure 1E). To evaluate the stability of circPTCH1, we treated the RNA from OS-RC-2 and A498 cells with RNase R and found that circPTCH1 was more stable to RNase R digestion whereas linear PTCH1 was clearly digested following RNase R treatment (Figure 1F). Similar results were observed using Actinomycin D (inhibitor of transcription) assay. As shown in Figure 1G, the half-life of the circPTCH1 transcript exceeded 24 h while linear PTCH1 transcription was blocked obviously. To investigate the subcellular localization of circPTCH1, we measured the expression of circPTCH1 in nuclear and cytoplasmic fractions of A498 and OS-RC-2 cells using qRT-PCR analysis and found that circPTCH1 was predominately distributed in the cytoplasm (Figure 1H). FISH assay also indicated that circPTCH1 was located predominantly in the cytoplasm (Figure 1I).

### circPTCH1 enhances the migration and invasion of RCC cells* in vitro*

**Figure 2:**
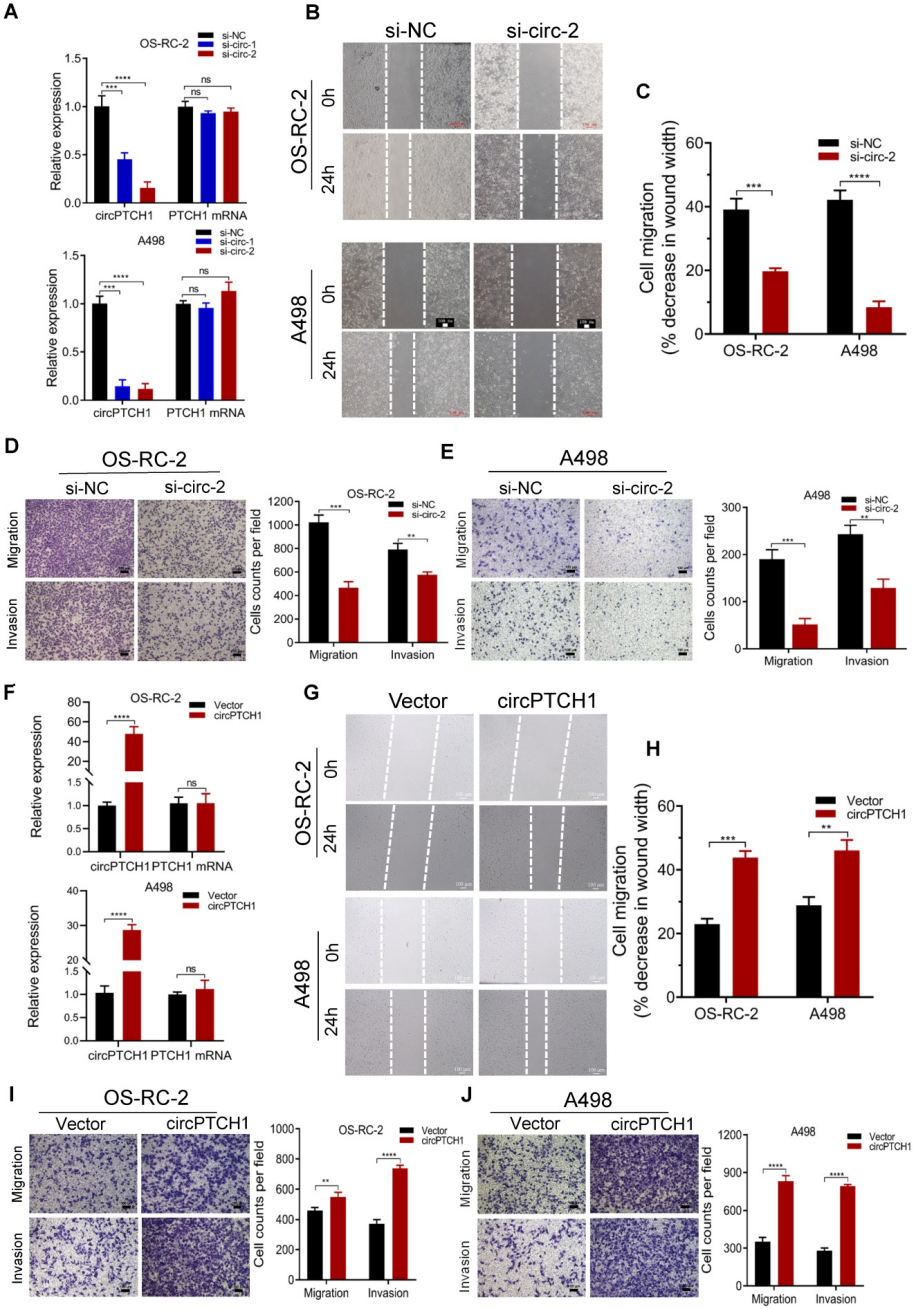
** circPTCH1 enhances the migration and invasion of RCC cells* in vitro*. A.** Two specific siRNAs (si-circ-1 and si-circ-2) were designed to interfere circPTCH1 expression and the suppressive efficacy on circPTCH1 and PTCH1 mRNA was measured by qRT-PCR. **B-C.** Cell migration capability of OS-RC-2 and A498 transfected with si-circ-2 or si-NC was assessed by wound healing assay. **D-E.** Cell migration and invasion abilities of OS-RC-2 and A498 transfected with si-circ-2 or si-NC was assessed by transwell migration and matrigel invasion assays. **F.** The expression of circPTCH1 and PTCH1 mRNA in OS-RC-2 and A498 cells transfected with circPTCH1 or vector plasmids were detected by qRT-PCR.** G-H.** Cell migration capability of OS-RC-2 and A498 transfected with circPTCH1 or vector was assessed by wound healing assay. **I-J.** Cell migration and invasion abilities of OS-RC-2 and A498 transfected with circPTCH1 or vector were assessed by transwell migration and matrigel invasion assays. Scale bar, 100 µm.*p < 0.05, **p < 0.01, ***p < 0.001. ****p < 0.0001. ns: none significance; NC: negative control; RCC: renal cell carcinoma.

To inhibit the expression of circPTCH1 in RCC cells, two siRNAs specifically targeting the junction sites of circPTCH1 were transfected into OS-RC-2 and A498 cells. Subsequent qRT-PCR results showed that both siRNAs (si-circ-1 and si-circ-2) decreased circPTCH1 expression but did not affect the PTCH1 mRNA levels (Figure 2A). Si-circ-2 was selected for use in latter assays as it has a better suppressive efficiency. Wound healing assays indicated that silencing circPTCH1 significantly suppressed cell migration in both OS-RC-2 and A498 cells (Figure 2B-C). Further, transwell assays also revealed that the migration and invasion of OS-RC-2 and A498 cells were blocked after inhibition of circPTCH1 (Figure 2D-E). Additionally, we upregulated circPTCH1 by transfecting the circPTCH1 overexpressed plasmids into RCC cells (Figure 2F). Conversely, the healing ability of A498 and OS-RC-2 cells was obviously enhanced (Figure 2G-H) and the migration and invasion of RCC cells were significantly increased in transwell migration and matrigel invasion assays (Figure 2I-J).

Next, we explored the correlations between circPTCH1 expression and special clinicopathological features in the set of 39 clinical RCC cases. As presented in Table 1, circPTCH1 expression was significantly positively related with RCC tumor Fuhrman grade and metastasis (*p* < 0.05).

In summary, the results from *in vitro* studies and clinical analyses indicate that circPTCH1 is significantly up-regulated in RCC tissues and cell lines and functions as an oncogene by promoting migration and invasion in RCC cells.

### circPTCH1 acts as a sponge of miR-485-5p in RCC cells

**Figure 3:**
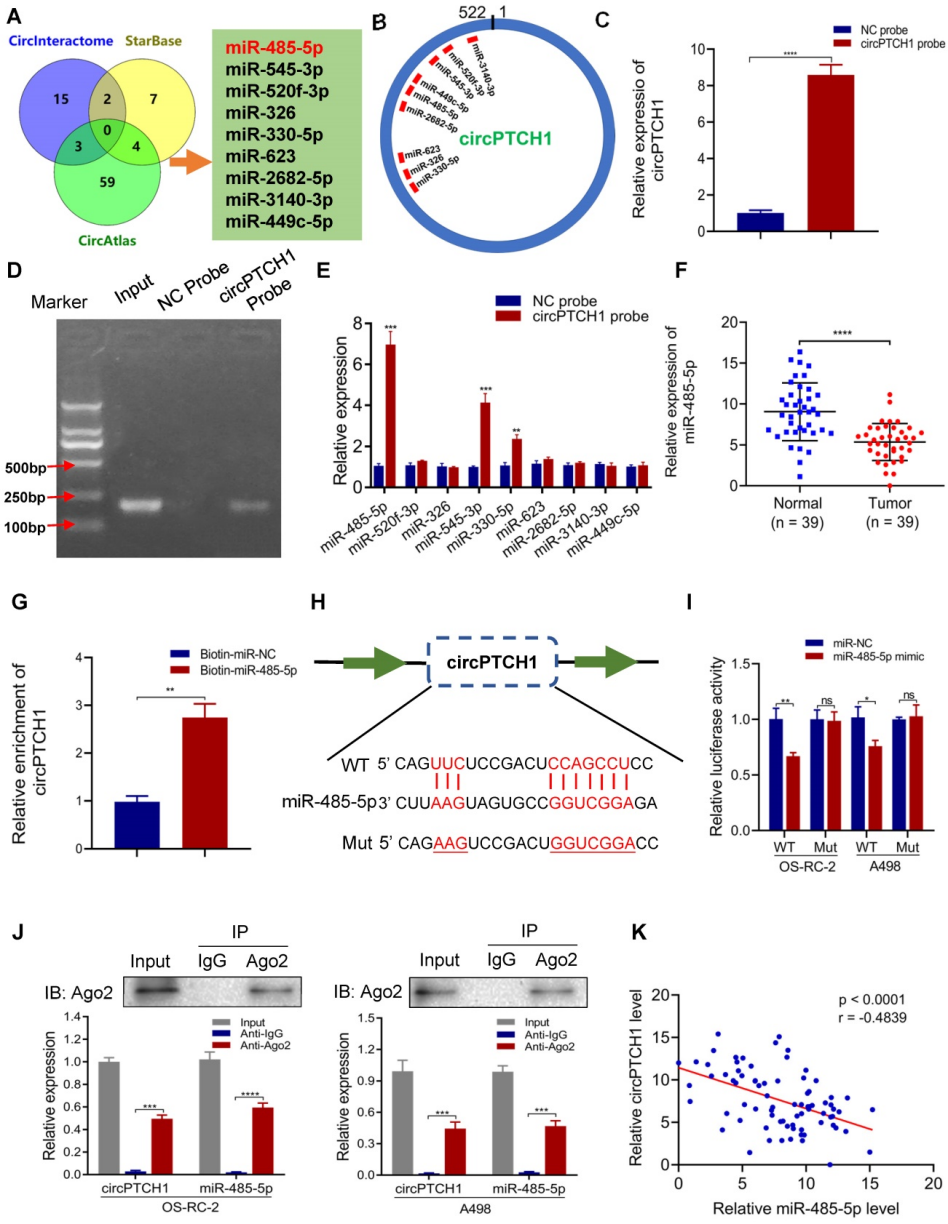
** circPTCH1 acts as a sponge of miR-485-5p. A.** Nine potential miRNAs absorbed by circPTCH1 were predicted through CircInteractome, Starbase and CircAtlas.** B.** Schematic model revealed the putative binding sites of nine miRNA candidates with circPTCH1. **C-D.** The efficiency of circPTCH1 probe was validated by qRT-PCR and gel electrophoresis. **E.** Relative expression of miRNAs enriched by circPTCH1 probe in HEK293T cells lysates was detected by qRT-PCR. **F.** The expression of miR-485-5p in 39 paired RCC tumor samples and adjacent normal tissues. **G.** Relative circPTCH1 level in HEK293T cells lysates captured by biotin-labeled miR-485-5p or NC was detected by qRT-PCR. **H-I.** Relative luciferase activities in RCC cells co-transfected with circPTCH1-WT or circPTCH1-Mut and miR-485-5p mimics or NC.** J.** RIP assay was utilized to verify the association between circPTCH1 and miR-485-5p. Top, IP efficiency of Ago2-antibody shown in western blotting. Bottom, relative RNA levels compared with input. IgG served as a negative control. **K.** circPTCH1 expression was inversely correlated with miR-485-5p level using Pearson correlation analysis in RCC samples. *p < 0.05, **p < 0.01, ***p < 0.001. ****p < 0.0001. ns: none significance; NC: negative control; Ago2: Argonaute-2; WT: wild type; Mut: mutant type; RCC: renal cell carcinoma; IP: Immunoprecipitation.

It has been reported that circRNAs, which mainly located in the cytoplasm, always function as a sponge of miRNAs 27. Thus, we predicted the potential target miRNAs of circPTCH1 using three online prediction tools: Circular RNA Interactome, circAtlas and starBase v2.0. In total, 90 miRNAs emerged as potential targets, but none were predicted across all three databases. Therefore, we narrowed the candidates to nine miRNAs (miR-485-5p, miR-545-3p, miR-520f-3p, miR-326, miR-330-5p, miR-623, miR-2682-5p, miR-3140-3p and miR-449c-5p) that were predicted across two of the three prediction tools (Figure 3A). The putative binding sites of circPTCH1 are shown in Figure 3B. RNA-pull down assay was performed to evaluate the interactions between these miRNAs and circPTCH1. Biotin-labelled circPTCH1 probe was synthesized by BIOFAVOR Biotech (Wuhan, China) and the efficiency was validated by qRT-PCR and gel electrophoresis (Figure 3C-D). Among these nine candidates, miR-485-5p, miR-545-3p, and miR-330-5p were abundantly pulled down by the circPTCH1 probe compared with the NC probe, and miR-485-5p was the most enriched (Figure 3E). Next, we investigated the expression of these miRNAs in RCC based on TCGA KIRC database. The results showed that only miR-485-5p was significantly down-regulated in RCC tissues (Figure S3), which implied that miR-485-5p may play the same anti-tumor role in RCC as previously reported in hepatocellular carcinoma and lung cancer 28, 29. To further validate this result, we measured miR-485-5p levels in our 39 paired RCC samples using qRT-PCR analysis. Indeed, miR-485-5p was significantly overexpressed in adjacent normal renal tissues relative to tumor specimens (Figure 3F). We next used biotin-labeled miR-485-5p mimics and NC to capture circPTCH1 and found that more circPTCH1 was captured by miR-485-5p mimics, further supporting the hypothesis that circPTCH1 acts as a sponge of miR-485-5p (Figure 3G).

To elucidate the interaction between circPTCH1 and miR-485-5p, we inserted the luciferase reporter with wild or mutant-type sequences that were predicted to be potential binding sites for circPTCH1 and miR-485-5p (Figure 3H). The relative luciferase activity was decreased by miR-485-5p mimics in OS-RC-2 and A498 cells transfected with wild-type constructs while no significant difference was observed in the mutant group (Figure 3I). Furthermore, we found that circPTCH1 and miR-485-5p could both be enriched by beads coated with anti-Ago2 compared with anti-IgG (Figure 3J). Additionally, as shown in Figure 3K, circPTCH1 expression was inversely associated with miR-485-5p levels in our clinical samples.

Taken together, the above results implied that circPTCH1 serves as a competing endogenous RNA (ceRNA) via sponging miR-485-5p.

### miR-485-5p suppresses the migration and invasion of RCC cells through targeting MMP14

**Figure 4:**
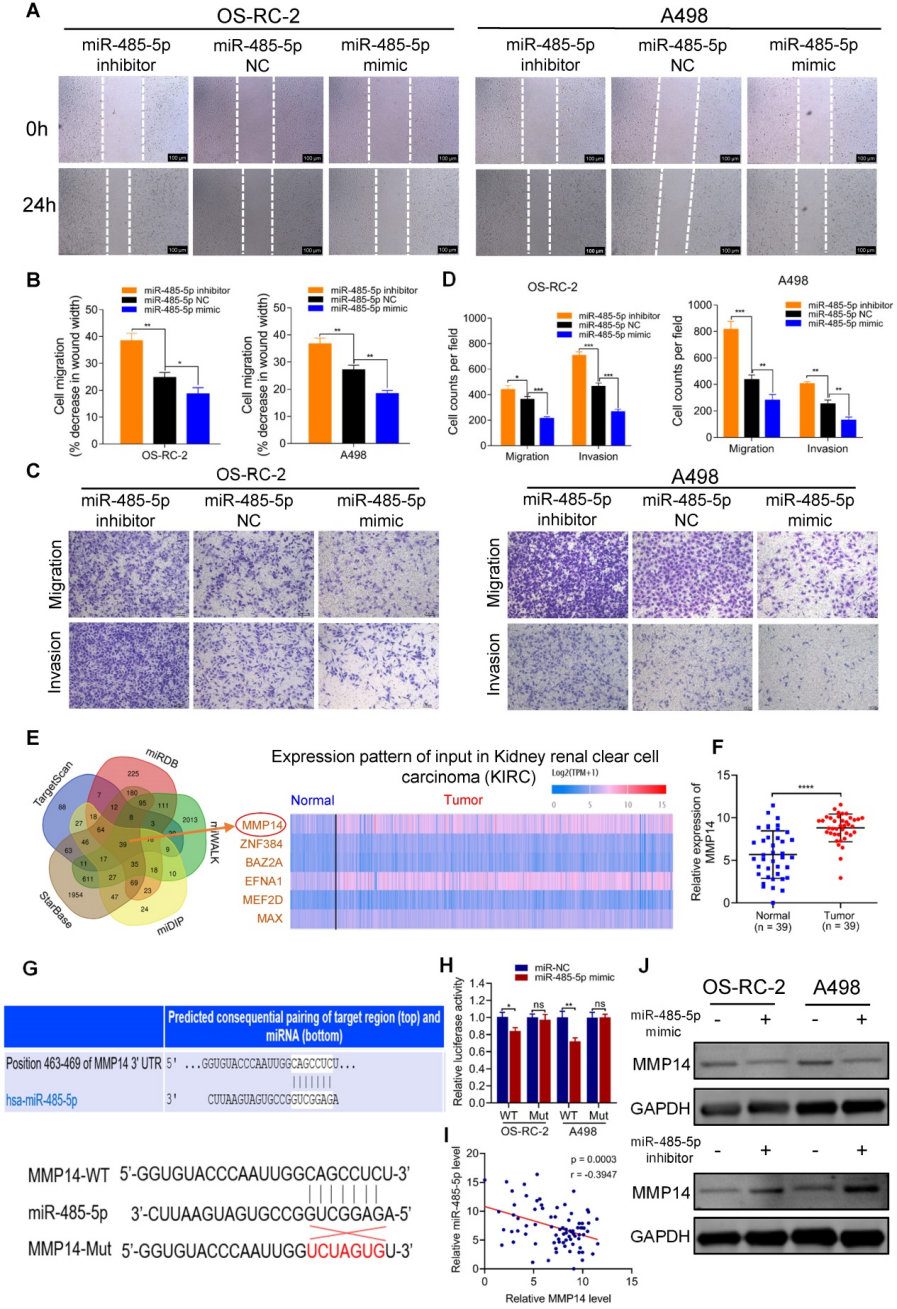
** miR-485-5p suppresses cell migration and invasion through targeting MMP14 *in vitro*. A-B.** Cell migration capability of OS-RC-2 and A498 transfected with miR-485-5p inhibitors, NC or mimics was evaluated by wound healing assay. **C-D.** Cell migration and invasion abilities of OS-RC-2 and A498 transfected with miR-485-5p inhibitors, NC or mimics were evaluated by transwell migration and matrigel invasion assays. **E.** Among the potential target genes of miR-485-5p predicted by bioinformatics analyses, six were included and exhibited in heatmap according to their expression in RCC samples based on TCGA KIRC database. **F.** The expression level of MMP14 in 39 paired RCC clinical samples evaluated by qRT-PCR.** G.** Schematic of MMP14 wild-type (WT) and mutant (Mut) luciferase reporter vectors. **H.** Relative luciferase activities were analyzed in RCC cells co-transfected with miR-485-5p mimics or miR-NC and WT or Mut luciferase reporter vectors. **I.** Correlation between miR-485-5p expression and MMP14 in 39 paired RCC clinical samples. **J.** The MMP14 protein levels in RCC cells were assessed by western blot after transfection with miR-485-5p inhibitors, NC or mimics. Scale bar, 100 µm. *p < 0.05, **p < 0.01, ***p < 0.001. ****p < 0.0001. NC: negative control; TCGA: the cancer genome atlas; KIRC: kidney clear cell carcinoma; RCC: renal cell carcinoma; WT: wild type; MUT: mutant.

To evaluate the role of miR-485-5p in RCC, we transfected miR-485-5p mimics, inhibitors, or NC into OS-RC-2 and A498 cells. These gain- and loss-of-function experiments showed that upregulation of miR-485-5p significantly inhibited the migration and invasion of OS-RC-2 and A498 cells, and silencing miR-485-5p enhanced the migration and invasion of RCC cells (Figure 4A-D).

To unravel the downstream target gene of miR-485-5p, we used a set of online prediction tools: TargetScan, starBase v2.0, miRDB, miWALK and miDIP. Initially, 39 candidates were identified across all prediction tools (Figure 4E). Then, we detected their expression in RCC tissues and found 6 (MMP14, ZNF384, BAZ2A, EFNA1, MEF2D and MAX) were significantly up-regulated in tumor tissues based on TCGA KIRC database and the Clinical Proteomic Tumor Analysis Consortium (CPTAC) ccRCC Dataset 30 (Figure S4A-B). Among those 6, we concentrated on MMP14 as it was the most aberrantly expressed (Figure S4A-B), and has been well-known relevant with metastasis of various tumors. Our clinical specimens also demonstrated that MMP14 was significantly differentially expressed in RCC samples (Figure 4F). Additionally, it has been reported that miR-485-5p suppresses migration and invasion of glioma cells through modulating the target gene MMP14 31. To validate the interaction between miR-485-5p and MMP14, we inserted the luciferase reporter with wild-type MMP14 3'-UTR or mutant sequences (Figure 4G). As shown in Figure 4H, the relative luciferase activity was efficiently decreased by miR-485-5p mimics in RCC cells transfected with wild-type constructs, while there was no significant difference observed in the MMP14 mutant group. Meanwhile, we observed a negative correlation between miR-485-5p and MMP14 expression in RCC samples (Figure 4I) and western blot showed that the protein level of MMP14 was altered following transfection with miR-485-5p inhibitors or mimics (Figure 4J), which also demonstrated that MMP14 is targeted by miR-485-5p.

We also assessed the prognostic value of miR-485-5p and MMP14 using the TCGA cohort. As shown in Figure S4C, high MMP14 expression correlated with worse patient survival (*p =* 0.018), consistent with our prior experiments. However, there was no apparent association between miR-485-5p and survival of RCC patients (*p =* 0.89) (Figure S4D). The relationship between expression of MMP14 or miR-485-5p and clinical features of RCC was also evaluated using TCGA data. As shown in Table S5, we found that MMP14 expression was significantly correlated with RCC tumor T stage (*p =* 0.014), grade (*p =* 0.044), and pathologic stage (*p =* 0.025). And miR-485-5p was observed significantly associated with RCC tumor T stage (*p =* 0.036). These results reveal that MMP14 or miR-485-5p may have potential as a novel prognostic indicator of RCC.

Collectively, these results demonstrate that miR-485-5p could significantly suppress migration and invasion of RCC through targeting MMP14.

### circPTCH1 facilitates migration, invasion, and EMT of RCC cells through miR-485-5p/MMP14 axis

**Figure 5:**
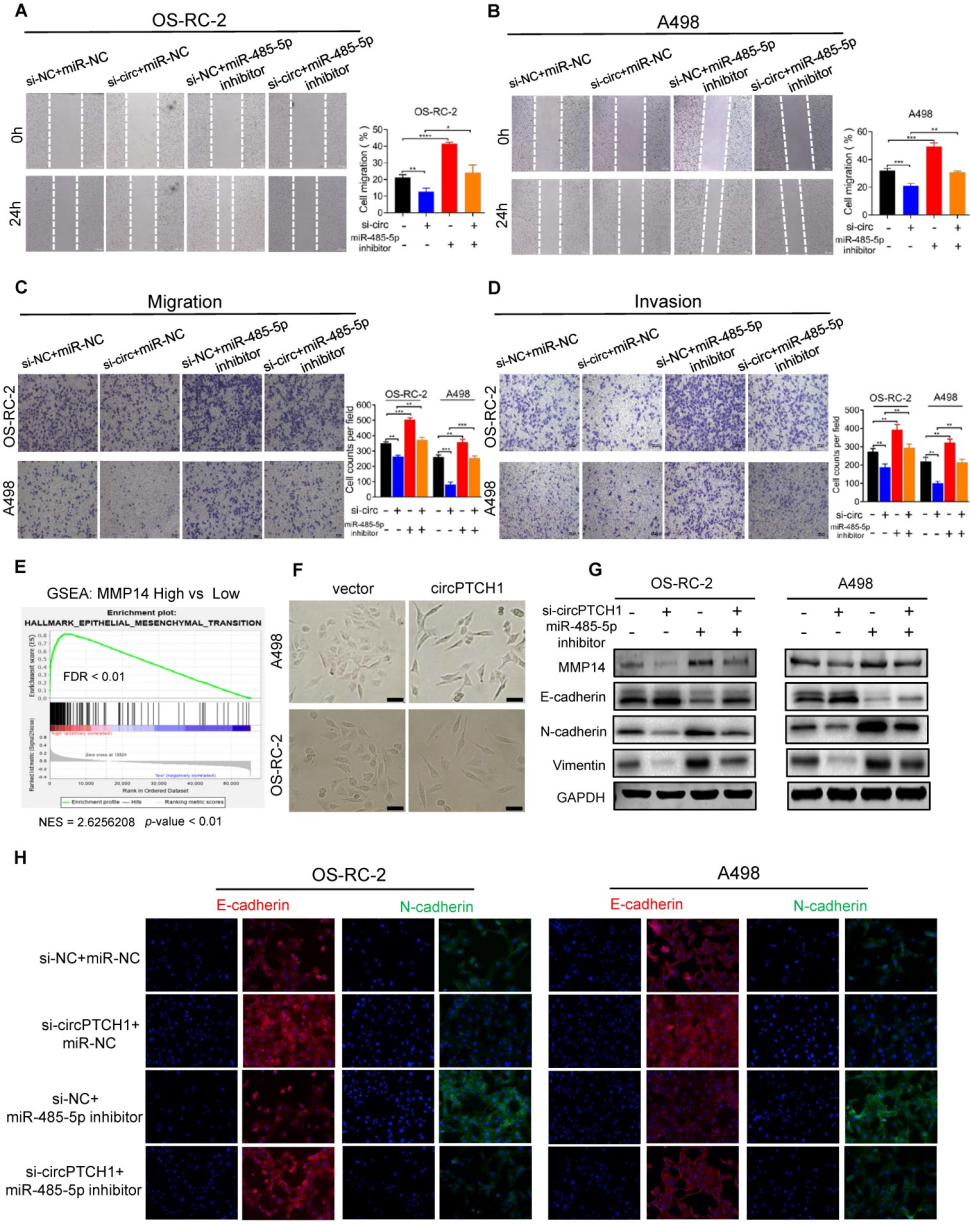
** circPTCH1 facilitates migration, invasion and EMT of RCC cells through miR-485-5p/MMP14 axis. A-D.** Wound healing and transwell assays showed the migration and invasion abilities of RCC cells after various treatments. **E.** GSEA analysis based on TCGA data showed that EMT was enriched in MMP14 overexpressed group. **F.** Bright field images of RCC cells morphology changes after overexpressing circPTCH1. Magnification × 100. **G.** The expressions of MMP14, epithelial marker (E-cadherin) and mesenchymal markers (N-cadherin and Vimentin) were detected by western blot.** H.** The expressions of E-cadherin and N-cadherin were detected by immunofluorescence analysis. Scale bar, 100 µm. *p < 0.05, **p < 0.01, ***p < 0.001. ****p < 0.0001. NC: negative control; EMT: epithelial-mesenchymal transition; RCC: renal cell carcinoma. GSEA: gene set enrichment analysis.

Rescue experiments were conducted to assess whether circPTCH1 promotes RCC migration and invasion through the miR-485-5p/MMP14 axis. The results of wound healing and transwell assays were consistent with previous data that attenuation of circPTCH1 decreased the migration of RCC cells. However, this effect was partly abolished after co-transfection with miR-485-5p inhibitors (Figure 5A-C). Similarly, the invasive abilities of OS-RC-2 and A498 cells were reversed with simultaneous inhibition of miR-485-5p (Figure 5D). The biological role of MMP14 was also determined using siRNA to silence its expression. Consistent with a previous study 32, MMP14 silencing suppressed migration and invasion of RCC cells and alleviated the pro-migration and -invasion effect of circPTCH1 on RCC cells (Figure S5). In addition, knockdown of circPTCH1 decreased the protein level of MMP14, while co-transfection with the miR-485-5p inhibitor partially restored its expression (Figure 5G).

EMT has been known as an important phenotypic indicator for tumor invasion and metastasis, by which epithelial cells acquire molecular alterations that lose their epithelial characterizations and obtain a mesenchymal feature 33. Here, we performed gene set enrichment analysis (GSEA) based on TCGA RCC dataset. As shown in Figure 5E, high MMP14 expression was associated with EMT (NES = 2.62; FDR < 0.01). Notably, morphological alterations of RCC cells were observed under light microscopy. After up-regulating circPTCH1 levels, a greater fraction of RCC cells gained a spindly, fibroblast-like morphology, which was consistent with characteristics of EMT (Figure 5F). We also measured the expression of several EMT pathway-related genes by western blot analysis. As expected, in circPTCH1-knockdown RCC cells, the expression of epithelial marker E-cadherin was increased and mesenchymal markers, N-cadherin and vimentin, were decreased (Figure 5G). Similar findings were also obtained using immunofluorescence analysis (Figure 5H). Together, the results of Figure 5A-H reveal that circPTCH1 promotes RCC migration, invasion and EMT through regulation of miR-485-5p/MMP14 signaling.

### circPTCH1 promotes tumor metastasis *in vivo*

**Figure 6:**
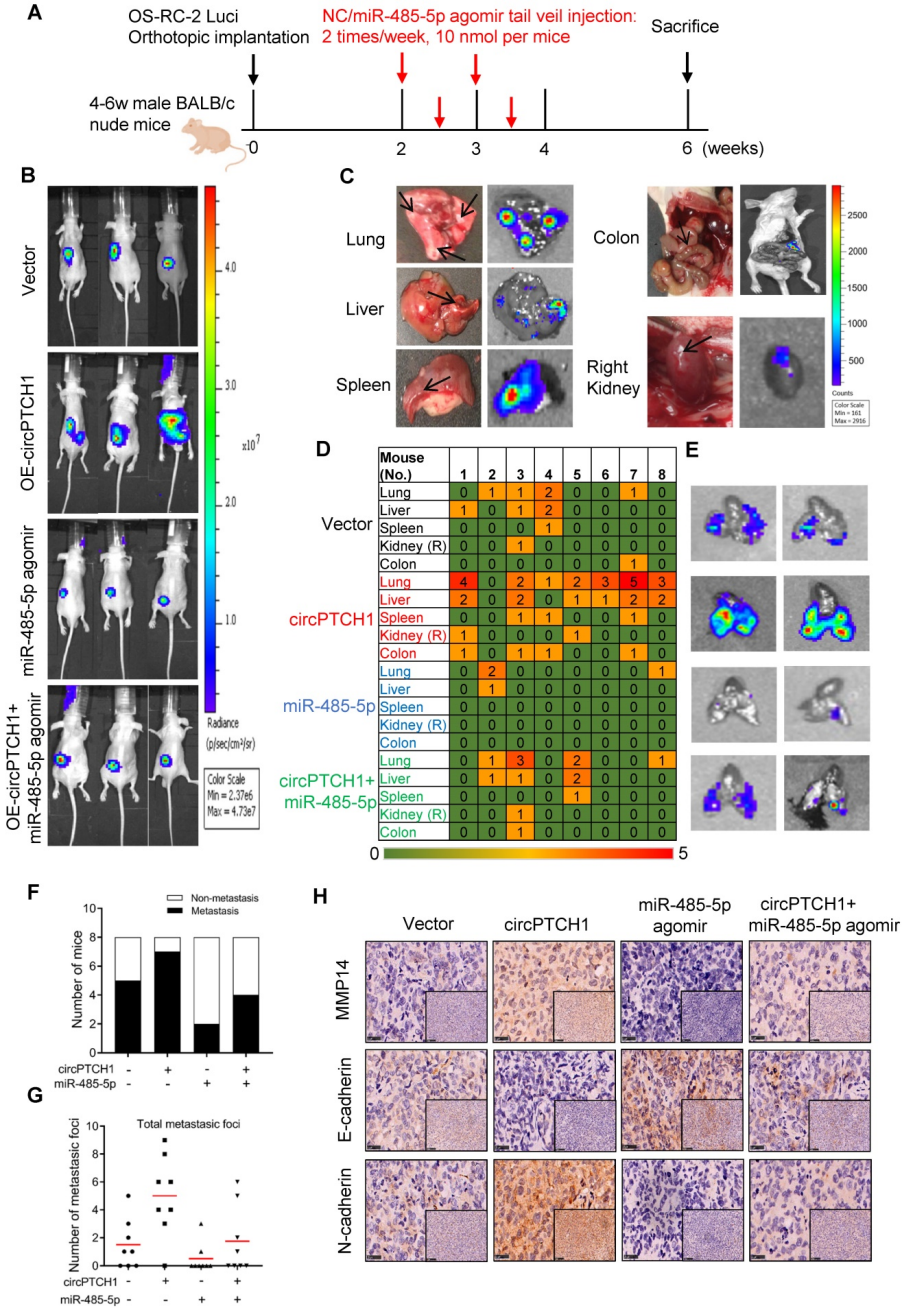
** circPTCH1 promotes tumor metastasis *in vivo*. A.** The flow diagram showed the scheme of tail vein injection with agomir miR-485-5p or NC into nude mice. **B.** IVIS imaging of mice 6 weeks after the orthotopically implant under the subrenal capsule of left kidney. Mice were distributed into 4 groups: vector, OE-circPTCH1, miR-485-5p agomir and OE-circPTCH1+miR-485-5p agomir (n = 8 each group). **C.** Representative photos and organ bioluminescent images of metastasis in lung, liver, colon, spleen, and the right kidney. **D.** Array diagram showed the metastatic status in various organs of each mouse in four groups. **E.** Representative bioluminescent images of lung metastases in four groups. **F.** Quantification of the metastasis rate in four groups. **G.** Total metastatic foci in each mouse of four groups.** H.** IHC detection of MMP14, E-cadherin and N-cadherin expression in tumor samples from nude mice in four groups. Magnification × 100 and × 400. NC: negative control; IHC: immunohistochemistry; IVIS: *in vivo* imaging system.

To further confirm the effect of circPTCH1 on RCC metastasis* in vivo*, we constructed stable circPTCH1 over-expressing cell line by infecting OS-RC-2 cells with circPTCH1-overexpressed lentivirus. The cells had been previously transfected with firefly luciferase 9. 1×10^6^ cells (circPTCH1-Vector or OE-circPTCH1) were injected into each mouse under the left subrenal capsule. The role of miR-485-5p on RCC metastasis was also investigated through tail vein injection with agomir-485-5p or NC. The injection scheme presented in Figure 6A was designed according to previous research 25. After 6 weeks, we found that mice in OE-circPTCH1 group developed more metastases compared with vector, and agomir-485-5p suppressed the progression of RCC and attenuated the effect of circPTCH1 (Figure 6B). The mice were then sacrificed and the total metastatic foci in lung, liver, colon, spleen and the contralateral kidney were counted using IVIS (Figure 6C-D). Elevated circPTCH1 expression led to an increased rate of metastasis as well as an increase in total metastatic foci and secondary metastatic lung tumor growth, while miR-485-5p inhibited this effect of circPTCH1 on RCC (Figure 6E-G). In parallel, IHC analysis was applied to detect MMP14, E-cadherin, and N-cadherin protein levels in tumor tissues from each group. Consistent with *in vitro* data, the expression of MMP14 and N-cadherin was up-regulated in the circPTCH1-OE group and down-regulated in the agomir-485-5p group, and the circPTCH1-induced increase in MMP14-EMT signaling was suppressed by injection of agomir-485-5p (Figure 6H). Taken together, these results indicate that circPTCH1 promotes RCC progression and metastasis via modulating miR-485-5p/MMP14/EMT signaling.

## Discussion

Numerous circRNAs have recently been identified with the development of high-throughput sequencing and advanced bioinformatics technology 34, and the relationship between circRNAs and cancers has drawn more attention than before. In this study, we reported a novel circRNA (circBase ID: hsa_circ_0139402) generated from exons 13 and 14 of the PTCH1 gene and evaluated its effect on RCC progression and metastasis.

**Figure 7:**
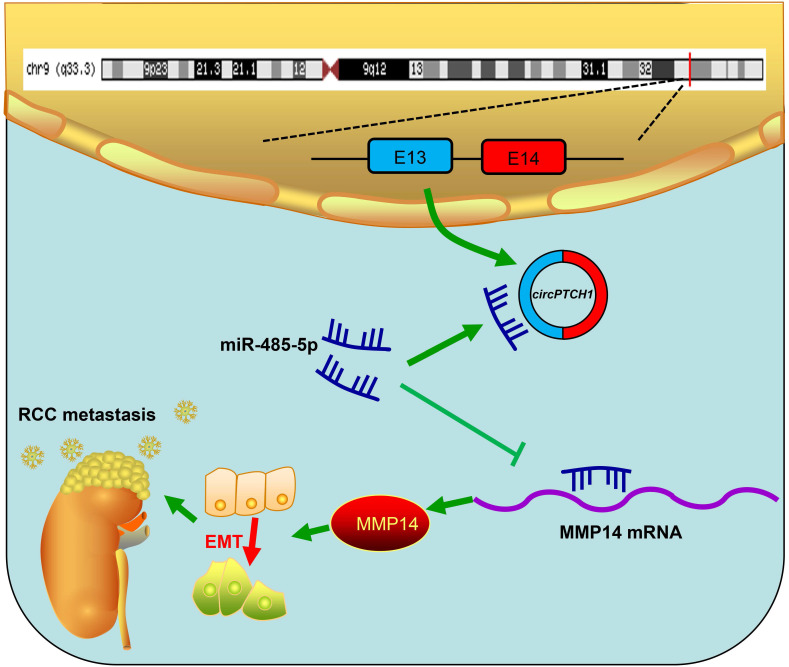
** Schematic diagram illustrates that circPTCH1 promotes RCC metastasis through miR-485-5p/MMP14 axis.** RCC: renal cell carcinoma; EMT: epithelial-mesenchymal transition; E: exon.

Although TKI and ICI drugs for metastatic RCC have been developed, the prognosis remains very poor, with a five-year survival rate less than 10% 2, 7. Thus, there is a significant need to clarify the mechanism of metastasis in RCC. In this study, we elucidated a new mechanism that RCC may develop metastasis ascribed to the dysregulated circPTCH1/miR-485-5p/MMP14 signaling (Figure 7). Our study demonstrated that circPTCH1 was up-regulated in the microarray analysis, tumor cell lines and clinical RCC samples, and high circPTCH1 levels were positively correlated with the degree of malignancy in patients with RCC. Upregulation of circPTCH1 stimulated RCC migration and invasion both* in vitro* and *in vivo* whereas inhibiting circPTCH1 reduced migration and invasion of RCC cells. Given the stability of circRNAs, they may represent a promising new type of diagnostic biomarker for cancers in the future. In the present research, we did not measure circPTCH1 levels in peripheral blood or urine of RCC patients due to the lack of samples. However, further studies need performed to investigate the expression of circPTCH1 in the circulation of RCC patients, which may indicate a newly and non-invasive method for RCC diagnosis.

Recently, circRNAs have been shown to be closely associated with tumorigenesis, such as cervical cancer 35, lung carcinoma 36, gastric cancer 37, hepatocellular carcinoma 38 and renal cell carcinoma. Xue *et al*. found that circ-AKT3 was lowly expressed in RCC tissues and suppressed the metastasis of RCC through activating the miR-296-3p/E-cadherin signaling pathway 39. circ-RAPGEF5 was reported to inhibit the growth and metastasis of RCC via the miR-27a-3p/TXNIP axis 40. circPRRC2A induces angiogenesis and metastasis through sponging miR-514a-5p and miR-6776-5p, and upregulating TRPM3 in RCC 41. Most of the studies on circRNAs have focused on the mechanism by which circRNAs function as a sponge of miRNA, thus influencing the target gene level. However, this competing endogenous RNA (ceRNA) effect is dependent on the cytoplasmic localization of circRNA and cannot be generally applied 42. It has been reported that circ-DONSON, which is mainly distributed in the nucleus, regulates the transcription of SOX4 by recruiting the NURF complex to its promoter 43, and circ-FBXW7, which is also located in the nucleus, represses glioma tumorigenesis through encoding a novel 21-kDa protein, FBXW7-185aa 44. In the present study, circPTCH1 was predominantly localized to the cytoplasm and acts as a sponge to competitively bind with miR-485-5p as demonstrated through RNA pull-down, RIP and dual-luciferase reporter assays.

MiR-485-5p has already been reported to suppress the progression of several tumors such as hepatocellular carcinoma, colorectal cancer and non-small cell lung cancer 18, 29, 45. However, its effect on RCC remained unknown. Here in this study, we firstly assessed the effect of miR-485-5p on RCC both *in vitro* and *in vivo*. The results demonstrate that miR-485-5p is aberrantly down-regulated in tumor specimens and could also suppress the migration and invasion of RCC cells.

MMP14 (also known as MT1-MMP), one of the zinc-dependent matrix metalloproteinase family, has been shown to promote EMT transition and induces invasive and metastatic activities in various tumors including RCC, through degrading extracellular matrix components (ECM) and several bioactive molecules 46-48. In the present study, MMP14 was predicted as the target of miR-485-5p by several online databases. Notably, the interaction between miR-485-5p and MMP14 has been investigated in previous publications. Bo *et al.* reported that LncRNA-MFI2AS1 promotes the growth, migration, and invasion of glioma cells by modulating MMP14 levels *via* miR-485-5p 31, and Cheng's study also implied that Lnc-UCA1 stimulates epithelial ovarian cancer through miR-485-5p/MMP14 signaling 49. Here, the interaction between miR-485-5p and MMP14 was confirmed by luciferase reporter analysis in RCC cells. Moreover, we observed that miR-485-5p expression level was inversely correlated with MMP14 in RCC clinical samples and the MMP14 protein levels were affected by transfection with miR-485-5p mimics or inhibitors, supporting the hypothesis that miR-485-5p targets MMP14.

In the present study, we explored the expression and function of circPTCH1 in RCC. However, the role of its host gene PTCH1, remains uncertain until now. One paper detected the immunohistochemical expression of PTCH1 in 140 ccRCC specimens and found that PTCH1 is more highly expressed in G3/G4 than in G1/G2 tumors (1.5-fold, *p =* 0.02) 50, which was consistent with our findings of circPTCH1. Another study evaluated the expression of PTCH1 in 37 ccRCC tumor and control tissues, however, no statistically significant differences were observed 51. Though circRNAs are typically described as having a similar function as their linear counterparts, antagonistic and independent functions of linear and circular RNA have also been reported previously. One study reported that circPOK functioned as a non-coding, proto-oncogenic RNA in mesenchymal tumors, in contrast to its linear gene Pokemon (functions as a tumor suppressor) 52. To clarify the molecular mechanism behind this role, the authors proposed and verified several hypotheses: sponging for miRNAs; encoding new proteins; and regulating the functionality of RNA-binding proteins through direct binding. Inspired by this study, we will investigate the role of PTCH1 in RCC and explore the underlying mechanisms in our future work.

In summary, this study indicates that circPTCH1 is highly expressed in RCC cells and tissues, and promotes RCC progression and metastasis by modulating MMP14 expression and activating EMT process via sponging miR-485-5p. These findings reveal a novel signaling pathway that may be applied as a potential prognostic biomarker and therapeutic target in metastatic RCC.

## Supplementary Material

Supplementary figures and tables.

## Figures and Tables

**Table 1:** The correlation between circPTCH1 level and various clinicopathologic features

Characteristics	Samples	circPTCH1 expression		*P* value
		Low	High	
Total	39	20	19	
**Age**				0.109
<60	22	11	11	
≥60	17	9	8	
**Gender**				0.504
Male	26	11	15	
Female	13	9	4	
**T stage**				0.118
1+2	33	18	15	
3+4	6	2	4	
**Metastasis**				0.032*
No	31	19	12	
Yes	8	1	7	
**Fuhrman grade**				0.045*
I+ II	30	18	12	
III+Ⅳ	9	2	7	

* Statistically significant.

## Abbreviations

RCC: renal cell carcinoma
KIRC: kidney clear cell carcinoma
ncRNA: non-coding RNA
FC: fold change
circPTCH1: circular RNA PTCH1
EMT: epithelial-mesenchymal transition
IHC: immunohistochemistry
qRT-PCR: quantitative real-time PCR
TCGA: the cancer genome atlas
CPTAC: clinical proteomic tumor analysis consortium
FISH: fluorescence *in situ* hybridization
RIP: RNA immunoprecipitation
siRNA: small-interfering RNA
WT: wild-type
MUT: mutant-type
PTCH1: patched 1
MMP14: matrix metallopeptidase 14
NC: negative control
DMEM: dulbecco's modified eagle's medium
FBS: fetal bovine serum
cDNA: complementary DNA
gDNA: genomic DNA
IVIS: *in vivo* imaging system
