# circPTCH1 promotes invasion and metastasis in renal cell carcinoma via regulating miR-485-5p/MMP14 axis: Erratum

**DOI:** 10.7150/thno.69378

**Published:** 2022-01-01

**Authors:** Huan Liu, Guanghui Hu, Zaoyu Wang, Qunlong Liu, Jin Zhang, Yonghui Chen, Yiran Huang, Wei Xue, Yunfei Xu, Wei Zhai

**Affiliations:** 1 Department of Urology, Shanghai Tenth People's Hospital, School of Medicine in Tongji University, Shanghai 200072, China.; 2 Department of Pathology, Renji Hospital, School of Medicine in Shanghai Jiao Tong University, Shanghai 200127, China.; 3 Department of Urology, Shanghai Tenth People's Hospital, Nanjing Medical University, Nanjing 210029, China.; 4 Department of Urology, Renji Hospital, School of Medicine in Shanghai Jiao Tong University, Shanghai 200127, China.

The authors regret that the image of OS-RC-2 invasion group was wrongly attached due to their carelessness in assembling figures (Fig.2I and Fig.5D) 1. The correct version is shown below.

The correction made in this erratum does not affect the original conclusions. The authors apologize for any inconvenience or misunderstanding that this error may have caused.

## Figures and Tables

**Figure 2:**
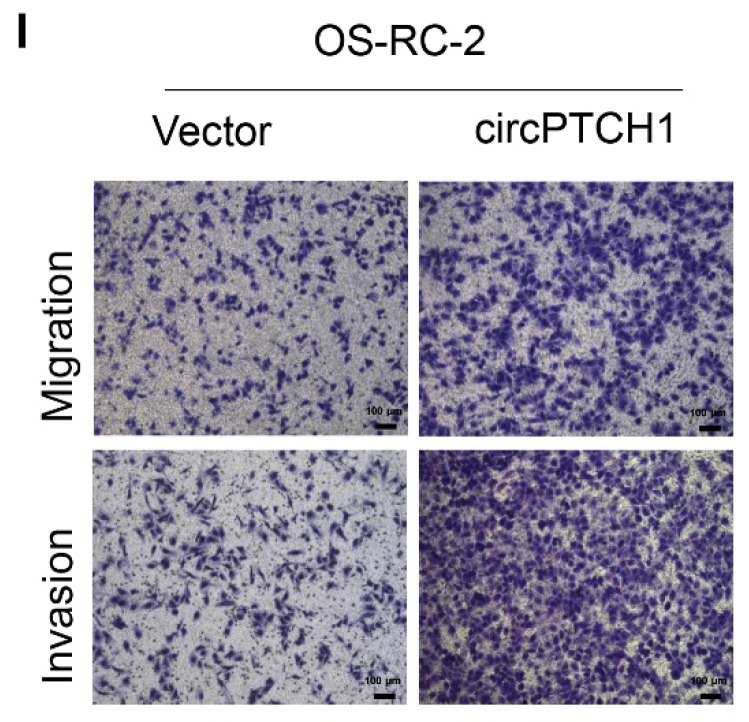
** I**: Cell migration and invasion abilities of OS-RC-2 transfected with circPTCH1 or vector were assessed by transwell migration and matrigel invasion assays.

**Figure 5:**
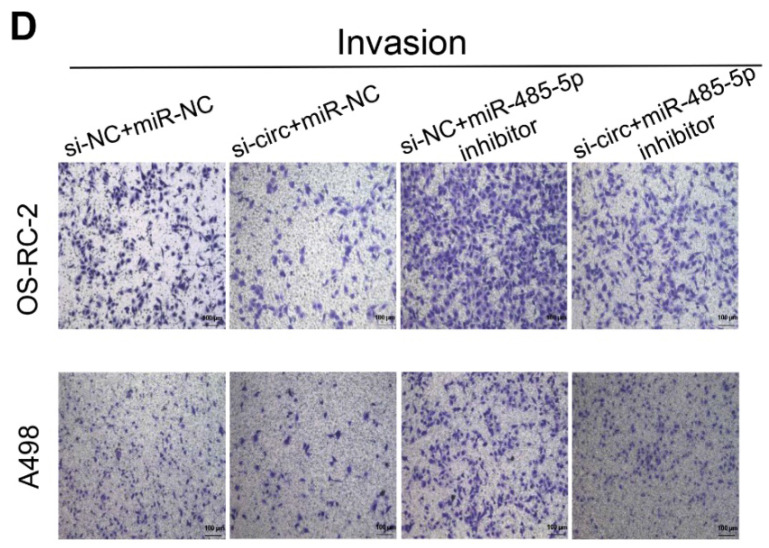
** D**: Transwell assays showed the invasion abilities of RCC cells after various treatments.
