# Supplementary material for: circPTCH1 promotes invasion and metastasis in renal cell carcinoma via regulating miR-485-5p/MMP14 axis
Source: Theranostics. 2020 Aug 29;10(23):10791–807. doi: 10.7150/thno.47239 (PMC7482820; doi:10.7150/thno.47239)
Supplement: Supplementary file 1 — Supplementary figures and tables. [file thnov10p10791s1.pdf]

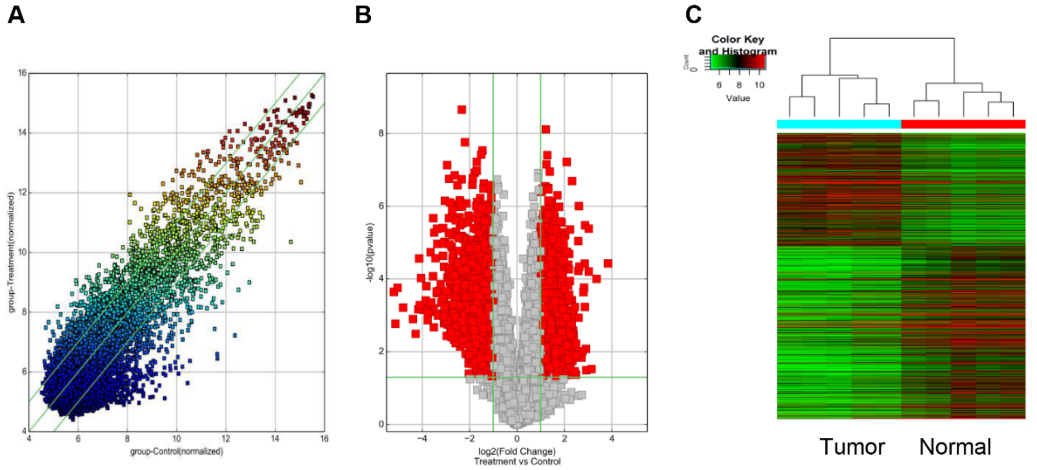

Supplementary figure 1. Microarray analysis of circRNAs from RCC patients' specimens. A: Scatter plot of microarray analysis. Green lines represent Fold Chang (FC). The dots above the top or below the bottom green line indicated circRNAs which | FC | >2.0. **B:** Volcano plot of microarray analysis. Red plots represent meaningful circRNAs (| FC | >2.0). **C:** Clustered heatmap of microarray analysis. Rows represent circRNAs and columns represent specimen types. RCC： renal cell carcinoma; FC: fold change.

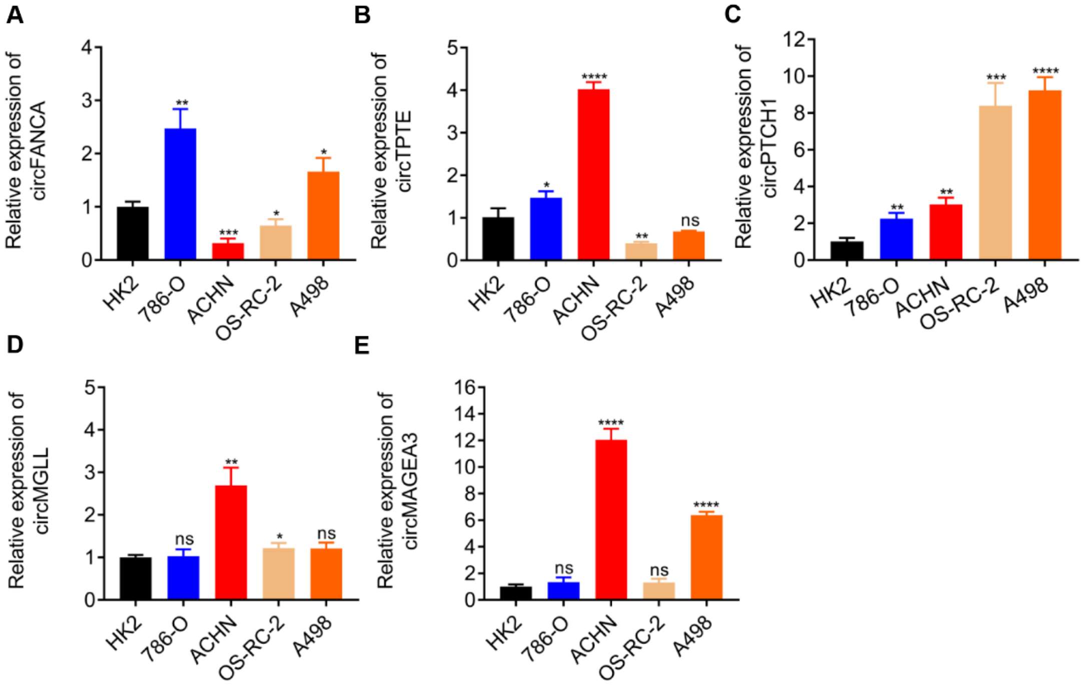

Supplementary figure 2. Relative expression of the top five up-regulated circRNAs in RCC cells compared to normal kidney tubular epithelial cell HK-2. A: circFANCA. **B:** circTPTE. **C:** circPTCH1. **D:** circMGLL. **E:** circMAGEA3. *$p < 0.05$, **$p < 0.01$, ***$p < 0.001$. ****$p < 0.0001$. ns: none significance.

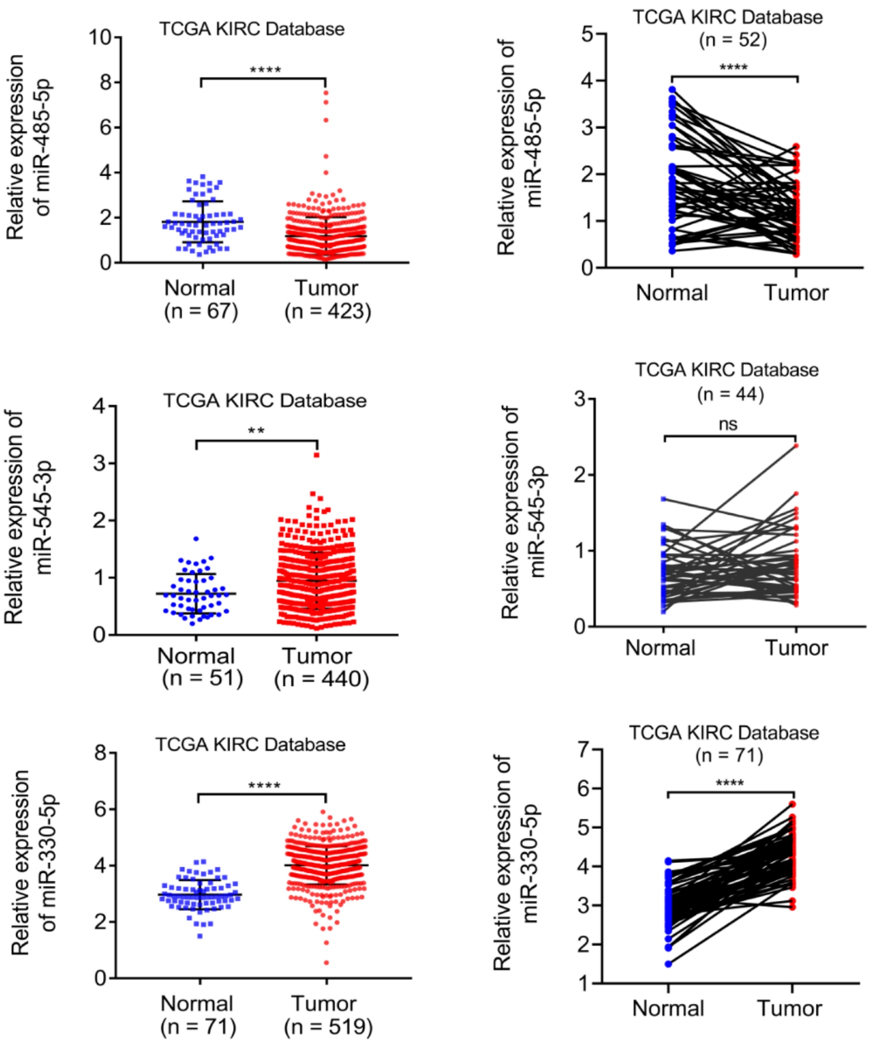

Supplementary figure 3. The expression of miR-485-5p, miR-545-3p and miR-330-5p in paired and non-paired RCC samples and normal tissues based on TCGA KIRC database. *$p < 0.05$, **$p < 0.01$, ***$p < 0.001$. ****$p < 0.0001$. ns: none significance. TCGA: the cancer genome atlas; KIRC: kidney clear cell carcinoma.

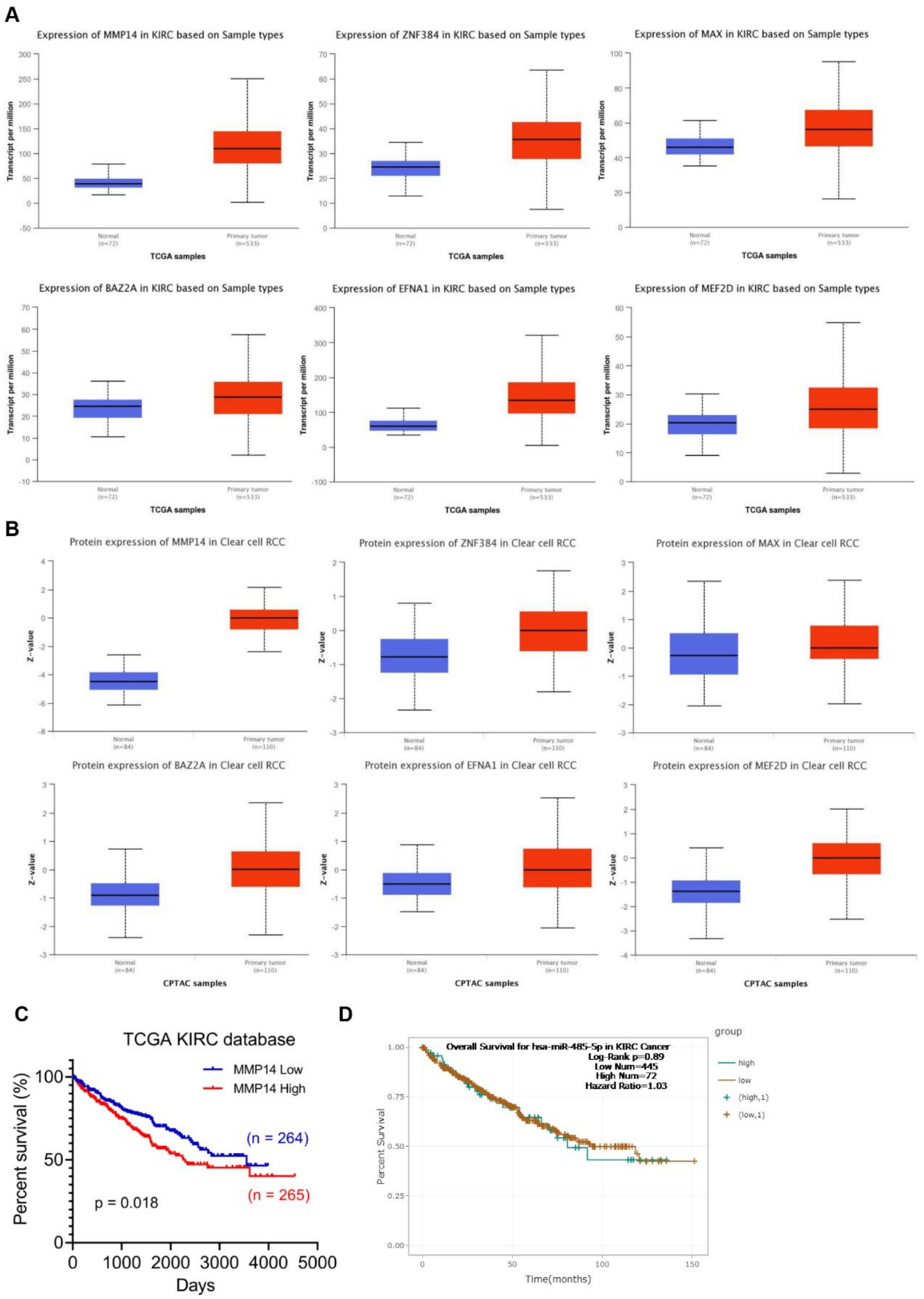

Supplementary figure 4. The expression and Kaplan-Meier's survival analysis of candidate genes. A: The expression level of six putative genes in RCC based on TCGA KIRC database. **B:** The expression level of six putative genes in RCC based on CPTAC Dataset. **C:** Kaplan-Meier's survival analysis of MMP14 using TCGA database. **D:**

Overall survival of miR-485-5p in KIRC cancer based on Starbase database. TCGA: the cancer genome atlas; KIRC: kidney clear cell carcinoma; CPTAC: clinical proteomic tumor analysis consortium.

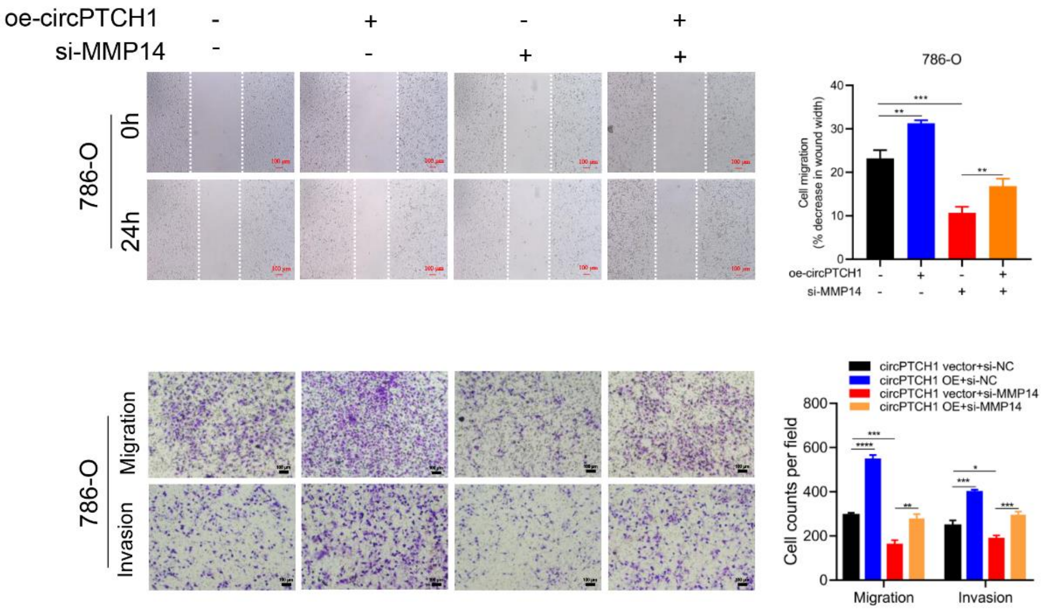

Supplementary figure 5. Abrogation of MMP14 alleviated the promotion of circPTCH1 on RCC cell migration and invasion *in vitro*. Scale bar, 100 µm. *$p < 0.05$, **$p < 0.01$, ***$p < 0.001$. ****$p < 0.0001$.

Supplementary Table 1:

Representative pathological figures of RCC cases in this study.

Renal cell carcinoma in situ:

patient 1:

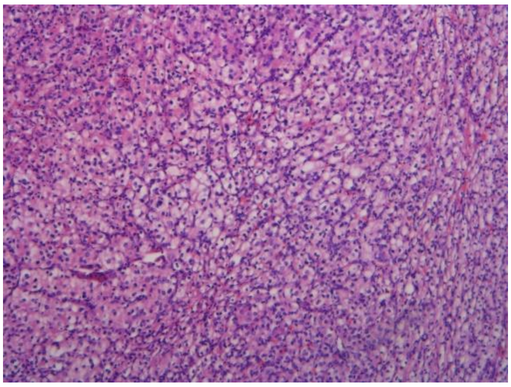

patient 2:

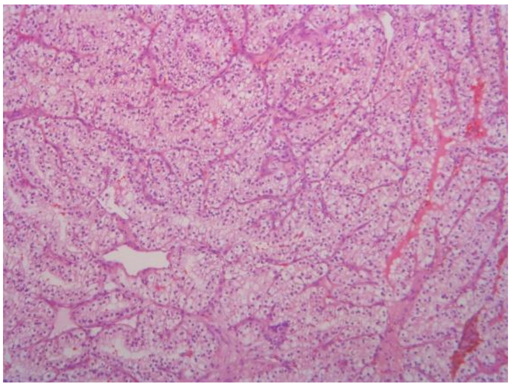

Lung metastasis:

patient 1:

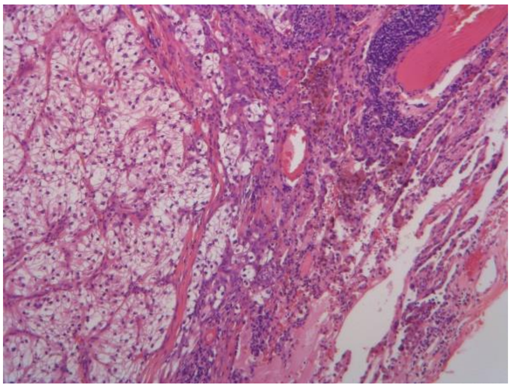

patient 2:

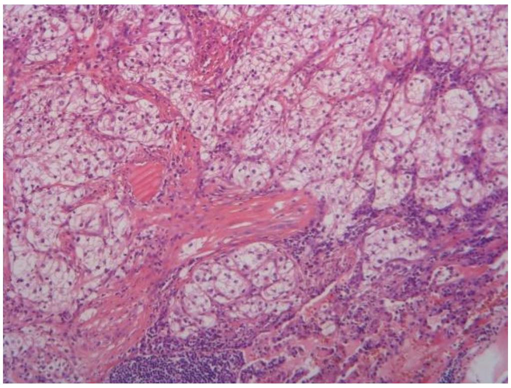

Bone metastasis:

patient 1:

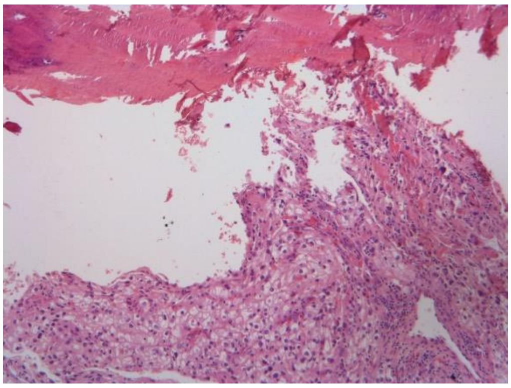

patient 2:

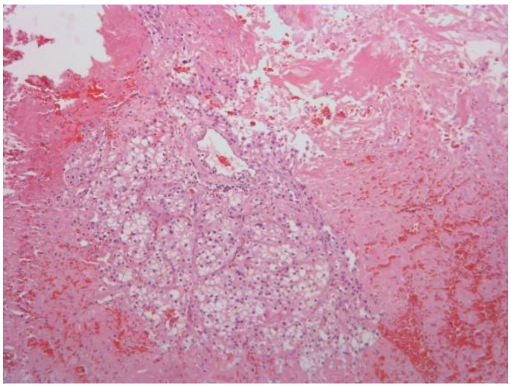

The clinicopathological features of the five metastatic RCC tissues used for circRNA microarray analysis.

Patient number	Age	Gender	Tumor	Fuhrman grade	Metastasis
1	59	Male	2b	II	Lung
2	47	Female	2a	II	Lung
3	62	Male	2a	III	Lung
4	69	Male	2b	III	Bone
5	64	Male	3a	II	Bone

The clinicopathological characterization details of 39 RCC patients.

Patient number	Age	Gender	Tumor	Metastasis	Fuhrman grade
1	68	Male	1a	0	I
2	66	Female	2a	0	III
3	59	Male	2b	1	II
4	54	Male	1a	0	I
5	51	Female	1a	0	I
6	56	Male	1a	0	II
7	69	Male	2b	1	III
8	62	Male	1b	0	II
9	54	Female	2a	1	II
10	58	Female	1a	0	I
11	62	Male	2a	1	III
12	52	Male	1a	0	I
13	73	Male	3a	0	II
14	59	Male	1a	0	II
15	37	Male	1a	0	II
16	50	Male	1a	0	I
17	51	Male	1b	0	II
18	67	Male	2a	0	III
19	64	Male	3a	1	II
20	51	Female	1a	0	I
21	49	Male	3a	0	II
22	54	Female	1b	0	I
23	58	Female	1a	0	I
24	62	Male	3a	1	II
25	71	Male	2a	0	III
26	56	Female	1b	0	II
27	69	Female	1b	0	II
28	76	Male	3a	1	II
29	57	Male	2a	0	II
30	52	Male	1a	0	II

31	58	Male	1a	0	I
32	61	Female	1b	0	II
33	65	Male	1a	0	I
34	52	Female	1a	0	I
35	66	Female	1b	0	I
36	74	Male	2a	0	II
37	47	Female	2a	1	II
38	69	Male	1b	0	II
39	49	Male	3a	0	II

Supplementary Table 2:
The website links and versions of online databases used in this study.

Bioinformatics analysis database	Websites
circBase	http://www.circbase.org/
Circular RNA Interactome	https://circinteractome.nia.nih.gov/
CircAtlas 2.0	http://circatlas.biols.ac.cn/
starBase v2.0	http://starbase.sysu.edu.cn/index.php
TCGA	https://xenabrowser.net/datapages/
UALCAN	http://ualcan.path.uab.edu/
TargetScan	http://www.targetscan.org/
miRDB	http://mirdb.org/
miWALK	http://mirwalk.umm.uni-heidelberg.de/
miDIP 4.1	http://ophid.utoronto.ca/mirDIP/

Supplementary Table 3:
Primers and RNA sequences used in this study

List of oligonucleotide sequences	5'--> 3'
**Primers for PCR**	
circFANCA Forward	TTCTTCTCCTTGATGGGCCTG
circFANCA Reverse	GTGGAAGAACTGCTCGCATC
circMEGEA3 Forward	ACGGATGGTTGAATGAGCGT
circMEGEA3 Reverse	CTTCTCCTTCAGTGCTCCTCC
circMGLL Forward	TTGCTGCGAAAGTGCTCAAC
circMGLL Reverse	GGCTCCATGGGACACAAAGA
circTAPE Forward	TGCCTGTTCTCAGTGTGGTC
circTAPE Reverse	ACTTTGATGCTCCAAATGGCAC
circPTCH1 Forward	ACCAAAAGCCAAGGCAGCGG
circPTCH1 Reverse	CCTCGCGTCGATATAAATCC
PTCH1 Forward	TTTTCTGCTGTTTTACAAGCCC
PTCH1 Reverse	CATGGTAATCTGCGTTTCATGG
MMP14 Forward	CAAGATTGATGCTGCTCTCTTC

MMP14 Reverse	ACTTTGATGTTCTTGGGGTACT
GAPDH Forward	CAATGACCCCTTCATTGACC
GAPDH Reverse	TTGATTTTGGAGGGATCTCG
hsa-miR-485-5p RT	GTCGTATCCAGTGCAGGGTCCGAGGTA TTCGCACTGGATACGACGCACACAA
hsa-miR-485-5p Forward	TGCGCTCAGCAAACATTTATTG
unified reverse primer	TGGTGTCGTGGAGTCG
U6 Forward	CTCGCTTCGGCAGCACA
U6 Reverse	AACGCTTCACGAATTTGCGT
**siRNAs**	
si-NC sense	UUCUCCGAACGUGUCACGUTT
si-NC antisense	ACGUGACACGUUCGGAGAATT
si circPTCH1-1 sense	CCAAAAGCCAAGGCAGCGGUATT
si circPTCH1-1 antisense	UACCGCUGCCUUGGCUUUUGGTT
si circPTCH1-2 sense	GAAACCAAAAGCCAAGGCAGCTT
si circPTCH1-2 antisense	GCUGCCUUGGCUUUUGGUUUCTT
si MMP14 sense	AACAGGCAAAGCUGAUGCAGATT
si MMP14 antisense	UCUGCAUCAGCUUUGCCUGUUTT
**RNA pull-down probes**	
NC probe	GAAGCACTATGCTCCTTTCCTCTTGAAA CCAAAAGCCAAGGCAGCGGTAGTAGTG GTGTTCAATTTTGCCATGG
circPTCH1 probe	CCATGGCAAAATTGAACACCACTACTA CCGCTGCCTTGGCTTTTGGTTTCAAGAG GAAAGGAGCATAGTGCTTC

RT: Reverse transcription; NC: Negative control; siRNA: small-interfering RNA.

Supplementary Table 4:
Top five upregulated circRNAs from microarray analysis

**Fold change**	**Regulation**	**circRNA**	**circBase name**	**Best_ transcript**	**Gene Symbol**
14.2815538	up	hsa_circRNA_ 101911	hsa_circ_0040994	NM_000135	FANCA
10.2219813	up	hsa_circRNA_ 103107	hsa_circ_0061251	NM_182482	TPTE
8.8333096	up	hsa_circRNA_ 407202	hsa_circ_0139402	NM_000264	PTCH1
8.141948	up	hsa_circRNA_ 103461	hsa_circ_0067185	NM_007283	MGLL
8.137467	up	hsa_circRNA_ 091722	hsa_circ_0091722	NM_005362	MAGEA3

Table S5: The correlation between MMP14/miR-485-5p level and RCC clinicopathologic features based on TCGA database.

Characteristics	Groups	Samples	MMP14 expression		P value	Samples	miR-485-5p expression		P value
			Low	High			Low	High	
**Total**		**491**	**246**	**245**		**374**	**187**	**187**	
**Age**	< 60	227	106	121	0.084	169	81	88	0.356
	≥ 60	264	140	124		205	105	100	
**Gender**	Female	166	85	81	0.749	130	63	67	0.434
	Male	325	161	164		244	122	122	
**T stage**	1+2	306	164	142	0.014*	231	107	124	0.036*
	3+4	185	72	113		143	80	63	
**Metastasis**	No	413	214	199	0.098	313	156	157	0.767
	Yes	78	32	46		61	29	32	
**Grade**	G1+G2	222	124	98	0.044*	165	91	74	0.148
	G3+G4	269	121	148		209	96	113	
**Stage**	I+ II	289	152	147	0.025*	216	113	103	0.086
	III+IV	202	93	109		158	74	84	

* Statistically significant
